# Tailoring the ligand shell for the control of cellular uptake and optical properties of nanocrystals

**DOI:** 10.3762/bjnano.6.22

**Published:** 2015-01-21

**Authors:** Johannes Ostermann, Christian Schmidtke, Christopher Wolter, Jan-Philip Merkl, Hauke Kloust, Horst Weller

**Affiliations:** 1 Institute for Physical Chemistry, University of Hamburg, Grindelallee 117, 20146 Hamburg, Germany; 2 Center for Applied Nanotechnology, Grindelallee 117, 20146 Hamburg, Germany; 3 The Hamburg Centre for Ultrafast Imaging, University of Hamburg, Luruper Chaussee 149, 22761 Hamburg, Germany; 4 Department of Chemistry, Faculty of Science, King Abdulaziz University, Jeddah, Saudi Arabia

**Keywords:** biolable, cellular uptake, fluorescence quenching, poylmeric micelles, quantum dots

## Abstract

In this short review, the main challenges in the use of hydrophobic nanoparticles in biomedical application are addressed. It is shown how to overcome the different issues by the use of a polymeric encapsulation system, based on an amphiphilic polyisoprene-*block*-poly(ethylene glycol) diblock copolymer. On the basis of this simple molecule, the development of a versatile and powerful phase transfer strategy is summarized, focusing on the main advantages like the adjustable size, the retained properties, the excellent shielding and the diverse functionalization properties of the encapsulated nanoparticles. Finally, the extraordinary properties of these encapsulated nanoparticles in terms of toxicity and specificity in a broad in vitro test is demonstrated.

## Introduction

One of the main challenges in using high quality nanoparticles for biological applications is to ensure that the ligand system surrounding the particles fulfills specific requirements [1]. On the one hand the ligands have to stabilize the particles at high dilution in aqueous media, be non-toxic and provide a good shielding against biological conditions (e.g., ions, peptides, enzymes). On the other hand the control over the size and functionality is desirable to study even these properties in the interaction between nanoparticles and biological material. Furthermore, the extraordinary properties of the nanoparticles should be retained during the phase transfer. Many different approaches for the phase transfer of hydrophobic nanoparticles have been described and analyzed concerning their behavior in biological media. Several excellent reviews of these methods can be found in the literature, very prominent and recent ones can be found in [2–3]. The here summarized phase transfer approach is based on amphiphilic diblock copolymers and meets most of the above mentioned requirements. Therefore amphiphilic diblock copolymers have been intensively discussed in drug delivery applications [4–6]. Due to the covalent connection of two chemically incompatible polymer blocks, these polymers show an interesting aggregation behavior in solvents, which are selective for only one of the blocks [7]. The formation of polymeric vesicles (polymerosomes) and spherical micelles is an interesting tool for the encapsulation of hydrophobic nanoparticles and since the critical micelle concentration (CMC) is comparatively low [8–9], a high stability against dilution can be achieved. By carefully choosing the monomers, reaction conditions and block length ratios it is possible to reproducibly synthesize very defined ligands, using anionic polymerization techniques [10]. One advantage of this reaction type is the absence of chain transfer and termination reactions, which gives the opportunity to easily functionalize the polymer chains using specific terminating reagents [11–12]. In this short review we summarize our experiences with amphiphilic diblock copolymers for the encapsulation of inorganic nanoparticles for their use in biomedical application and show how the cellular response can be tuned by tailoring the molecular properties of the polymer ligands.

A typical hydrophilic polymer ligand which is used for the coating of nanoparticles [13–14] and in the drug application [15] is poly(ethylene glycol) (PEG). PEG has already proven to be non-toxic and to minimize unspecific interactions with the immune system, resulting in enhanced blood circulation times of so-called PEGylated drugs [16–18]. For the second block, polyisoprene is a suitable material due to its incompatibility with PEG, which ensures a good formation of self-assembled vesicles and micelles in water, depending on the chosen block length ratio [19]. The high amount of present double bonds in the micelle core offers the possibility of radically initiated cross-linking of the structures or even microemulsion polymerizations to produce very dense capsules.

## Review

### Ligand synthesis

**Scheme 1:**
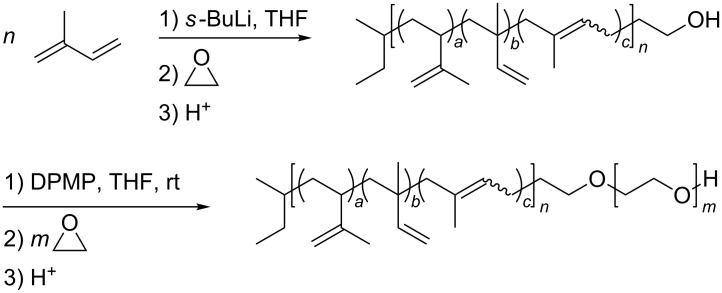
Reaction scheme of the PI-*b*-PEG diblock copolymer synthesis.

The synthesis of amphiphilic polyisoprene-*block*-poly(ethylene glycol) (PI-*b*-PEG) was realized via anionic polymerization, using standard high vacuum reaction techniques [10,20]. Isoprene was polymerized in dry THF by the quick addition of *sec*-butyllithium at −40 °C. After completion of the reaction, the living anion was functionalized by the addition of ethylene oxide and subsequent quenching with acetic acid to obtain an alcohol functionalized polyisoprene (PI). In the following step, the terminal hydroxy group was deprotonated with diphenyl methyl potassium (DPMP) to obtain a solvate separated ion pair, which is capable of starting the polymerization of ethylene oxide at room temperature (Scheme 1). Using this reaction path a variety of PI homopolymers and PI-*b*-PEG diblock copolymers differing in size and block length ratio were synthesized (Table 1).

**Table 1:** Analytical data of synthesized PI-*b*-PEG diblock copolymers.

Name	*M*_n_^a^	*M*_w_^a^	*M*_w_/*M*_n_^a^	wt % PEG^b^

PI-*b*-PEG 1	2200	2800	1.27	42
PI-*b*-PEG 2	2700	3000	1.11	51
PI-*b*-PEG 3	4300	4700	1.07	68
PI-*b*-PEG 4	5900	6300	1.07	70
PI-*b*-PEG 5	6600	8100	1.22	73
PI-*b*-PEG 6	9000	9900	1.09	65
PI-*b*-PEG 7	10000	11300	1.13	75
PI-*b*-PEG 8	10500	11900	1.13	67
PI-*b*-PEG 9	13400	14300	1.07	70
PI-*b*-PEG 10	13700	14200	1.04	65

^a^By SEC measurements, using PEG standards. ^b^Calculated by NMR measurements.

**Figure 1:**
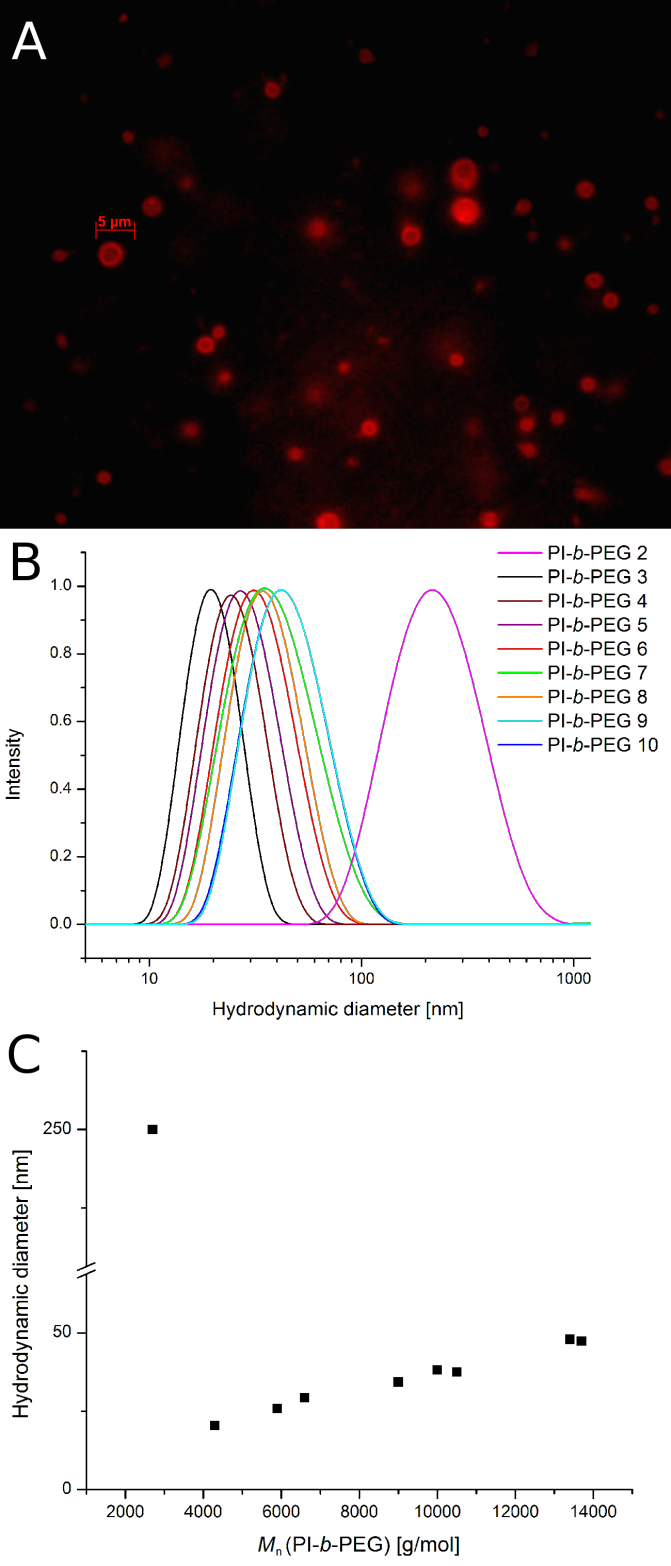
Fluorescence microscopy image of vesicles from PI-*b*-PEG 1 in water, the bilayer was visualized using the hydrophobic dye Nile Red (A); Size distribution of the micelles build from PI-*b*-PEG 2 – PI-*b*-PEG 10 in water, determined by DLS measurements (B); Correlation between the molecular weight of the PI-*b*-PEG and the build micelles (C).

The aggregation of the PI-*b*-PEGs in water was investigated and showed the expected behavior depending on the size and block length ratio of the polymer [19]. The analytical data of the used characterization methods like dynamic light scattering (DLS) and fluorescence microscopy are summarized in Figure 1. As can be seen from the microscope image, the PI-*b*-PEG with 42 wt % PEG forms unilamellar vesicles in water in a size range between 2 and 5 µm. The sample containing 51 wt % PEG shows a big hydrodynamic diameter of about 250 nm with a broad size distribution in the DLS measurements (Figure 1B). This could be attributed to the formation of cylindrical or wormlike micelles, especially if these results are compared to the measurements of the other PI-*b*-PEG aggregates in water, which show much smaller hydrodynamic diameters with a good correlation between the polymer and the micelles size (Figure 1C).

The CMC values of the polymers were determined based on the varying fluorescence properties of pyrene, depending on the polarity of the surrounding medium [21]. The values correlate well with the polymer size and lie between 6.0 × 10^−7^ M for the smallest and 1.5 × 10^−7^ M for the biggest polymer.

### Encapsulation methods for nanoparticles

As shown before a multiplicity of PI-*b*-PEG ligands can be synthesized using living anionic polymerization. In this part the focus is set on the use of different PI-*b*-PEGs and the effect of these different molecules on the physical properties of the obtained nanocontainers. The encapsulation of inorganic nanoparticles has been shown to depend on several parameters, like the ratio between ligands and particles or the surface chemistry of the particles. In this review the discussion is limited to the encapsulation of highly fluorescent QDs in PI-*b*-PEG for the use in biological systems. Since cellular uptake mechanisms except from phagocytosis are known to work best with small structures below 150 nm [22], only spherical micelles fulfilling this requirement will be discussed.

**Figure 2:**
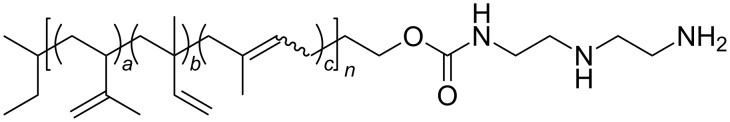
Illustration of the diethylene-triamine functionalized PI (PI-DETA).

To ensure a good compatibility between the hydrophobic particles and the inner core of the final micellar container, a partial ligand exchange with a diethylene-triamine functionalized PI (PI-DETA; see Figure 2) is conducted, usually in the range of 1000 g/mol up to 3000 g/mol, but also bigger PI-DETA have already been used successfully.

After the PI coating, the QDs are dissolved in THF, mixed with the PI-*b*-PEG and are injected into a 10-fold excess of water, followed by heating to 80 °C for several hours to remove the THF. Depending on the required density of the nanocontainer, a radical initiator can be added to partially cross-link the double bonds of the PI core. The addition of the radical initiator has shown to drastically enhance the fluorescence properties, namely the fluorescence quantum efficiency, of encapsulated CdSe/CdS/ZnS and CdSe/Cd_x_Zn_(1-x)_S/ZnS core–shell–shell quantum dots (QDs). This enhancement can be explained to a certain extent by the cross-linking of the micelles but further investigations showed a radical mediated annealing of the particle surface and crystal structure leading to a more crystalline ZnS shell with less defects [23].

**Figure 3:**
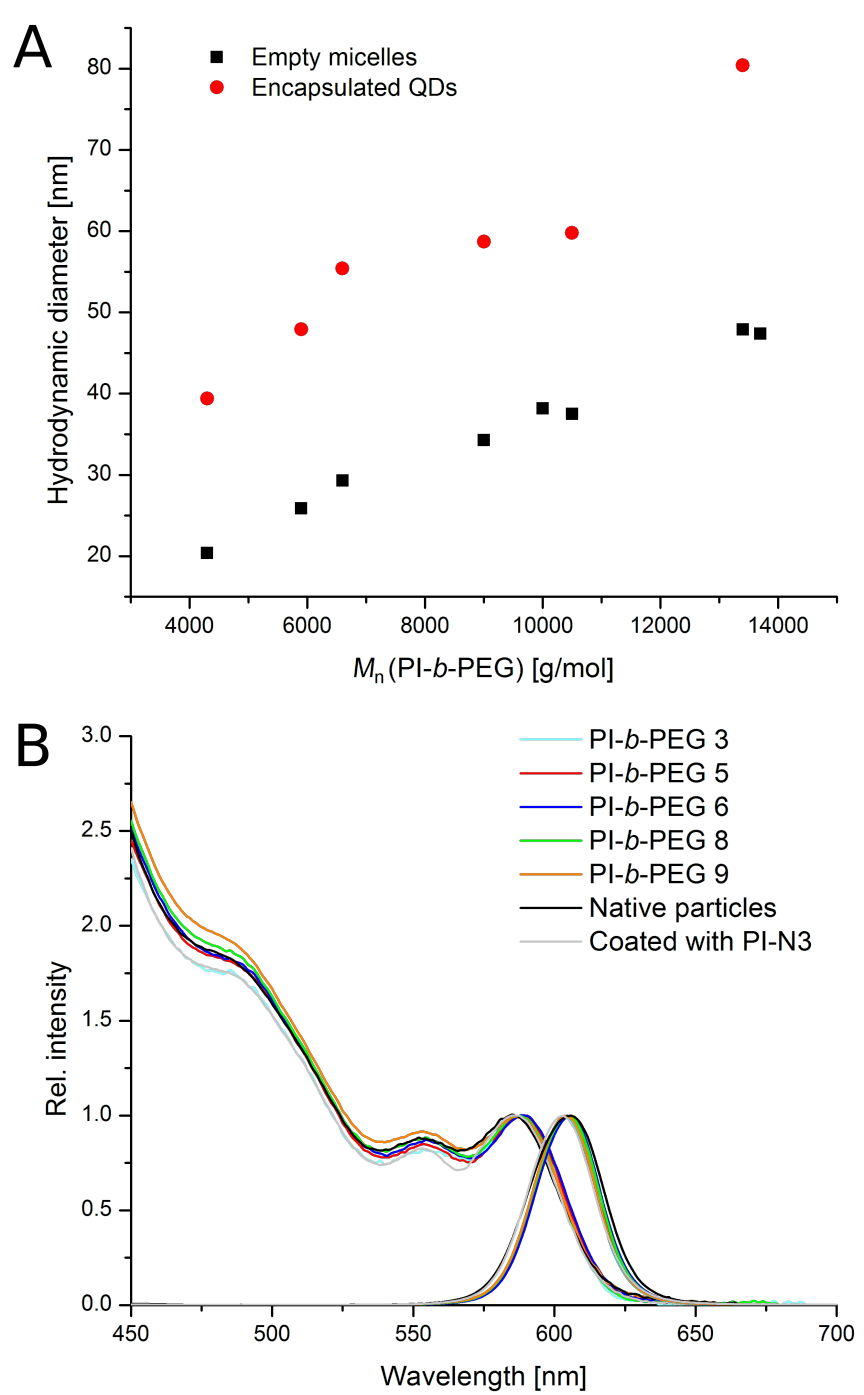
Conserved correlation between the molecular weight of the PI-*b*-PEG and the size of empty micelles (black squares) and encapsulated QDs (red dots) (A); Absorption and emission spectra for native and PI-DETA coated QDs in organic and QDs encapsulated in different PI-*b*-PEGs (B) [24].

The presented micellar encapsulation process worked very well and reproducibly with all PI-*b*-PEG diblock copolymers independently of the molecular weight. The fluorescence properties like the characteristic narrow emission band or the absorption maximum of the QDs did not change due to the phase transfer, as can be seen from Figure 3B. In fact, it was not only possible to conserve the particles properties, but to also to keep the correlation between the molecular weight of the polymer and the hydrodynamic diameter of the final construct containing the fluorescing QDs. The associated results from DLS measurement are shown in Figure 3A.

**Figure 4:**
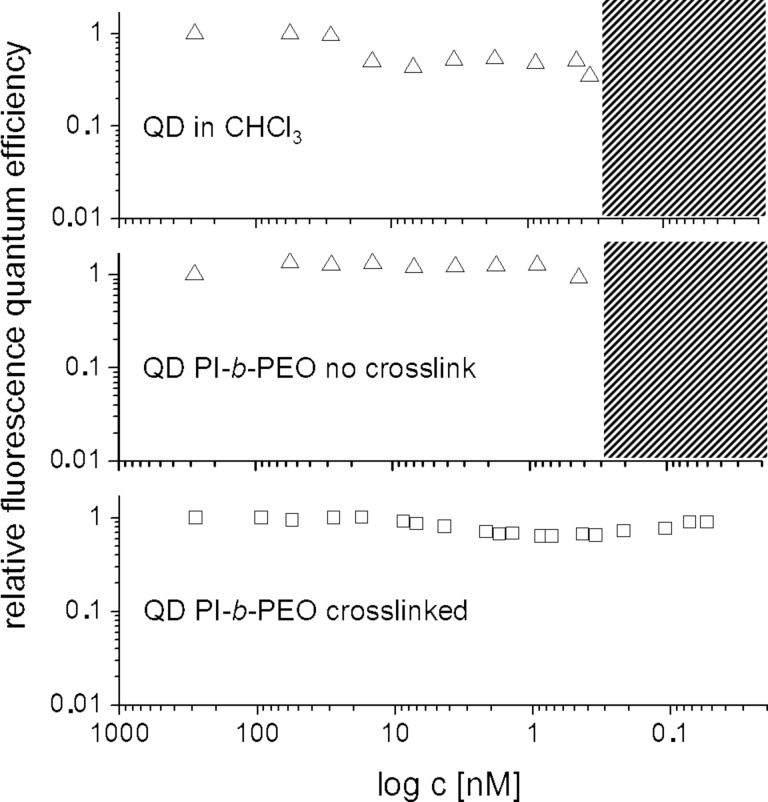
Fluorescence quantum efficiency in dependence of the dilution. At the top for native QDs in CHCl_3_, in the middle for QDs in non-cross-linked micelles and at the bottom the increased stability against dilution for the QDs in cross-linked micelles. Reprinted with permission from [25]. Copyright 2012 American Chemical Society.

The enhanced stability of the cross-linked nanocontainers was proven by dilution experiments, in which native QDs in chloroform, QDs in a regular PI-*b*-PEG micelle in water and QDs in a cross-linked micelle in water were stepwise diluted until the fluorescence of the particles ceased due to aggregation or the collapsing of the ligand system. The particles in cross-linked micelles showed an increased stability against dilution, which was a magnitude higher than for the regular micelles and the native ligands as can be seen in Figure 4 [25]. Furthermore cross-linked micelles hindered ion-accessibility on QDs as is discussed in the following section.

**Figure 5:**
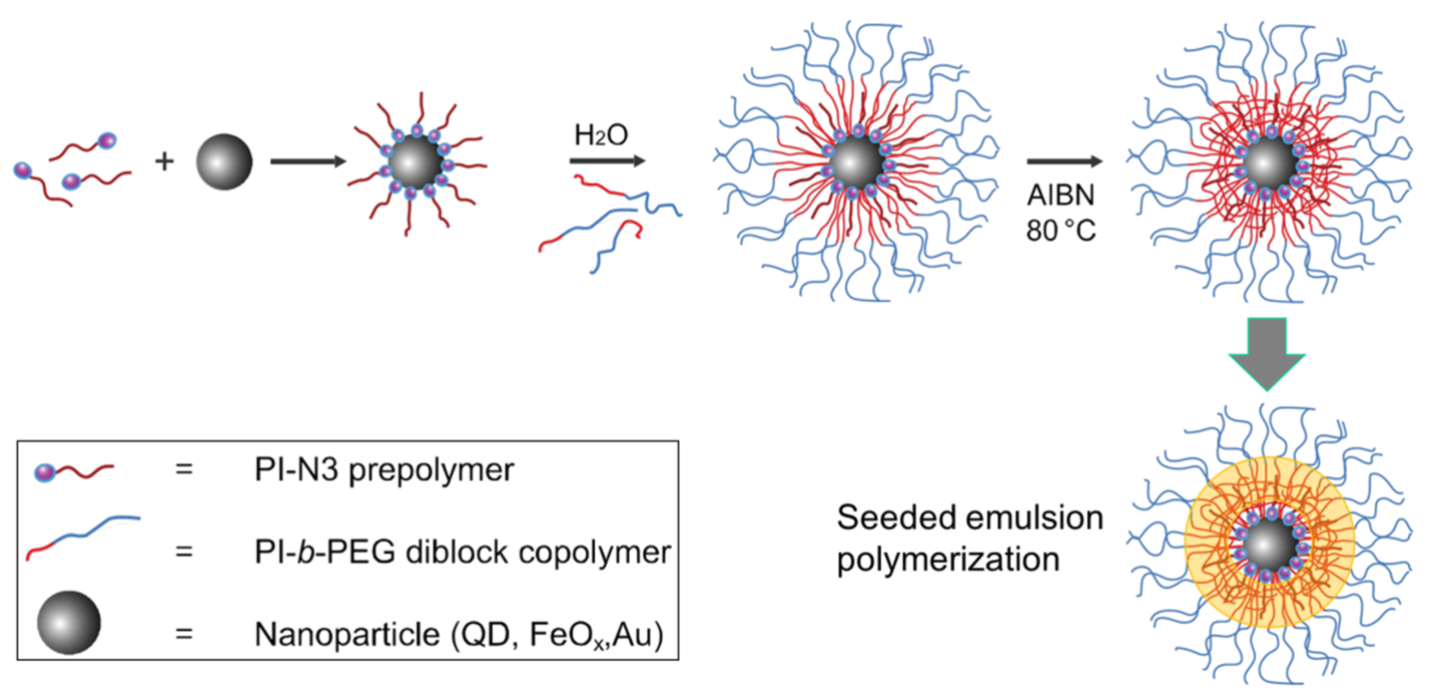
Schematic presentation of the encapsulation of inorganic nanoparticles in a nanocontainer, based on PI-*b*-PEG including optional cross-linking and seeded emulsion polymerization steps for a higher density of the core region. Reproduced with permission from [27]. Copyright 2013 The Royal Society of Chemistry.

To produce an even denser hydrophobic core region a seeded emulsion polymerization has been developed, in which additional monomer like styrene, isoprene and divinylbenzene are added as a cross-linking reagent after the micellar encapsulation and polymerized in the hydrophobic core [26]. The monomers react with the double bonds of the PI forming a covalently coupled dense shell. Figure 5 shows the whole encapsulation process, including the possible cross-linking and seeded emulsion polymerization steps.

A very powerful and sensitive tool to test the density of the nanocontainers is fluorescence quenching with small organic molecules or ions to simulate biological conditions. The big variety of options before, during and after the encapsulation has a strong influence on the properties of the final nanocontainer. As for the stability against dilution, the radically initiated cross-linking has a positive influence on the shielding properties of the micelle around the encapsulated nanoparticles. This can be seen from the experiments performed by Schmidtke et al. in which iron(III) ions and methylviologene were added stepwise to a solution of cross-linked and non-cross-linked QDs. Fluorescence quenching was less pronounced in the case of cross-linked micelles indicating hindered diffusion towards the QD and therefore a denser shell [23]. A strong enhancement of the density could also be observed in the case of QDs stabilized by emulsion polymerization [26].

**Figure 6:**
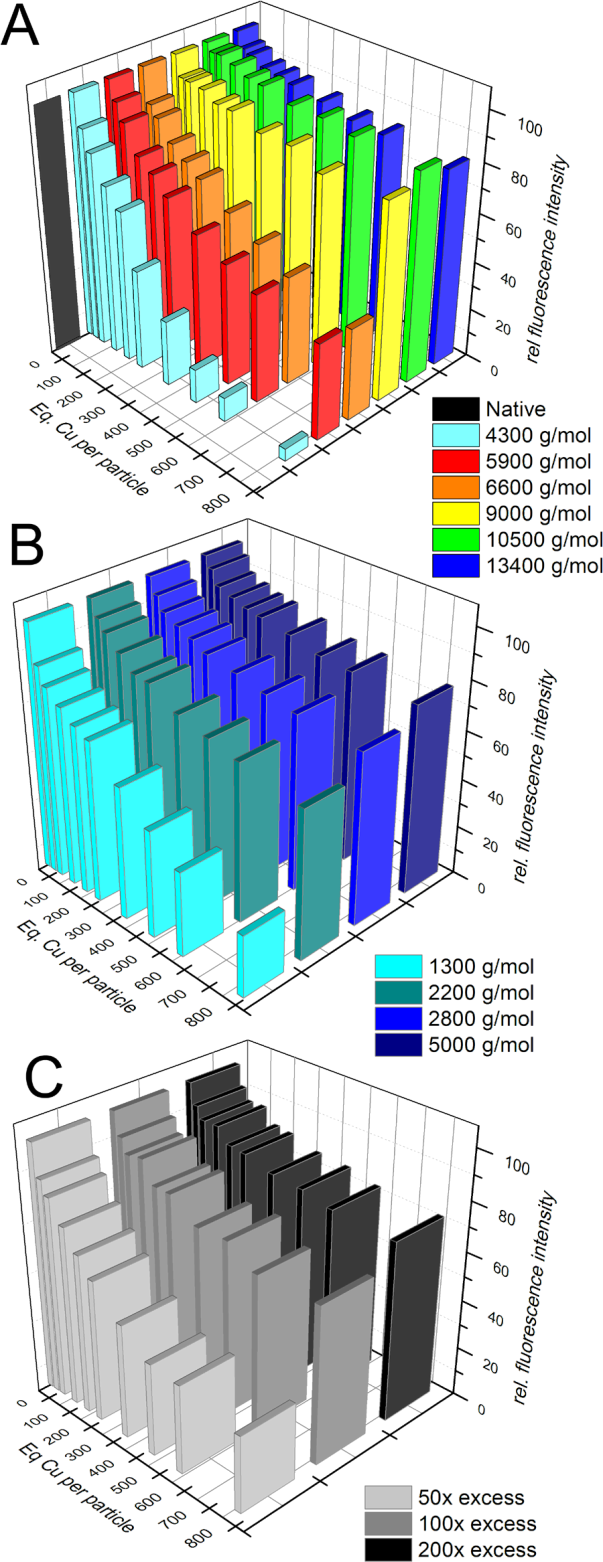
Relative fluorescence intensity of QDs after the addition of aliquots of Cu^2+^. Coated with a 1300 g/mol PI-DETA and encapsulated in PI-*b*-PEG of different molecular weights (A) (Reprinted with permission from [24]. Copyright 2013 American Chemical Society); coated with differently sized PI-DETA and encapsulated in a PI-*b*-PEG with a *M*_N_ of 5900 g/mol (B); coated with a 1300 g/mol PI-DETA and encapsulated in a PI-*b*-PEG of 6600 g/mol, using different excesses (C) during phase transfer (Figure reproduced with permission from [28]. Copyright 2014 Society of Photo Optical Instrumentation Engineers).

Finally, the used polymers and ratios between QDs and the polymer have a strong influence on the shielding against ions, as can be seen in Figure 6. By using the differently sized PI-*b*-PEG diblock copolymers for the encapsulation of QDs a strong correlation between the polymer size and the density of the micellar core can be observed [24]. Since the hydrophobic region is thought to have the biggest influence on the shielding, samples using differently sized PI-DETA ligands, but the same mid-sized PI-*b*-PEG (5900 g/mol) were produced. Again, a strong correlation between the size of the PI-DETA and the shielding can be observed, which confirms the assumptions concerning the importance of the hydrophobic core region. Finally, the amount of ligand molecules per QD has to be chosen carefully. Merkl et al. showed the importance of a properly chosen ligand to QD ratio in fluorescence quenching experiments, using a 6600 g/mol PI-*b*-PEG. A clear trend can be observed as the fluorescence is less quenched, the more ligands are present around the particles, although this trend is only valid to a certain extent of ligand excess [28]. It has to be noted, that these experiments were performed, using different types of QDs so the results are not directly comparable. However, the trends for all three experiments are very strong and reproducible. Further results obtained from quenching experiments based on PEG- and PI-*b*-PEG ligand systems, including a branched polymer of the type PI-*b*-(PEG)_2_ can be found elsewhere [13,24,28–29].

Taking advantage of the drastically increased shielding against quenching ions like copper, first experiments using copper catalyzed azide–alkyne cycoladdition (Click chemistry) have proven the possibility to functionalize PI-*b*-PEG encapsulated QDs, retaining their fluorescence properties [26,30]. This superior property opens new paths in functionalization possibilities for this type of nanocontainers additional to the subsequently presented strategies.

### Functionalization properties

For the biological use of encapsulated nanoparticles functionalizing of the capsules with relevant molecules (sugars, peptides), proteins or DNA is required. The most common coupling strategies are based on specific chemical groups like amines, carboxyl and hydroxyl functions [31]. Functional groups are not only useful for typical coupling strategies of biomolecules, but also for the determination of the surface properties of the final nanocontainers. Differently charged particles show different unspecific interactions with cells, like macrophages, epithelic or endothelic cells [32]. For macrophages the internalization process follows the typical steps of phagocytosis, which is controlled by the adsorption of specific proteins on the surface of the nanocontainer. Hydrophobic and charged particles in general (positively or negatively) show a much more efficient adsorption of these proteins needed for the recognition by macrophages [33–34]. Other cell types follow the endocytotic process, which can be receptor mediated or unspecific. For the uptake via endocytosis a positive surface charge has shown to enhance the cellular uptake, due to the attractive interaction with the negatively charged cell membrane [35–36]. Therefore, control over the surface chemistry is crucial to study the nanocontainers behavior in vitro and in vivo.

**Figure 7:**
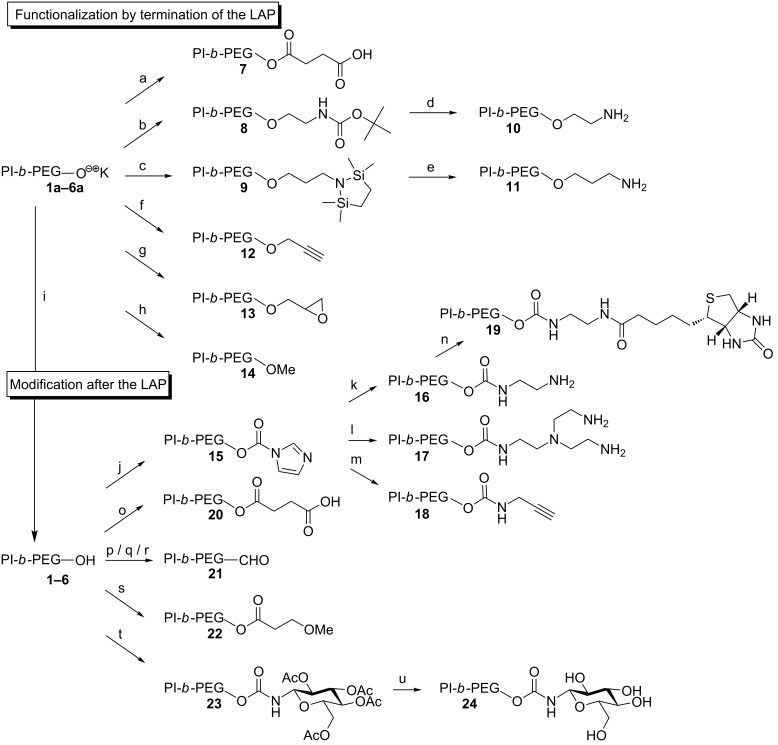
Synthetic routes of the modified PI-*b*-PEG ligands. (a) succinic anhydride, THF; (b) 2-(boc-amino)ethyl bromide, THF; (c) 1-(3-bromopropyl)-2,2,5,5-tetramethyl-1-aza-2,5-disilacyclopentane, THF; (d) 1 M HCl, THF; (e) HCl, THF; (f) propargyl bromide, THF; (g) epibromohydrin, THF; (h) methyl iodide, THF; (i) CH_3_COOH, THF; (j) CDI, DABCO, CH_2_Cl_2_; (k) ethylenediamine, DABCO, CHCl_3_; (l) tris(2-aminoethyl)amine, CHCl_3_; (m) propargylamine, DABCO, CHCl_3_; (n) biotin *N*-succinimidyl ester, DABCO, DMF, THF; (o) succinic anhydride, DABCO, CHCl_3_; (p) acetic anhydride, DABCO, CHCl_3_, DMSO; (q) DMP, H_2_O, CHCl_3_; (r) PDC, CH_2_Cl_2_; (s) 3-methoxypropionyl chloride, THF; (t) 2,3,4,6-tetra-*O*-acetyl-β-D-glucopyranosyl isocyanate, DABCO, CHCl_3_; (u) NaOMe, MeOH, Amberlite^®^ IR120 (hydrogen form). Reproduced with permission from [30]. Copyright 2013 The Royal Society of Chemistry.

Figure 7 shows possible functionalization of PI-*b*-PEG prior to the encapsulation of nanoparticles which were realized so far [30]. These functionalization reactions can be subdivided into two different types, the functionalization by termination of the anionic polymerization and the modification after the anionic polymerization.

By the termination of the living alkoxide with α-halogen or succinic anhydride compounds several useful groups for subsequent coupling strategies are easily accessible in a Williamson ether synthesis. Using this approach, carboxy-, amine-, alkyne-, epoxide- and methoxide-functionalized PI-*b*-PEG could be obtained. The typical termination with acetic acid leads to the formation of hydroxy-functionalized PI-*b*-PEG, which can be further functionalized by known procedures for the functionalization of PEG [37–38]. Via this approach PI-*b*-PEGs bearing a biotin and a sugar as first examples for small biological molecules were accessible. A detailed overview about glycol-conjugated strategies using click chemistry is provided in [39].

**Figure 8:**
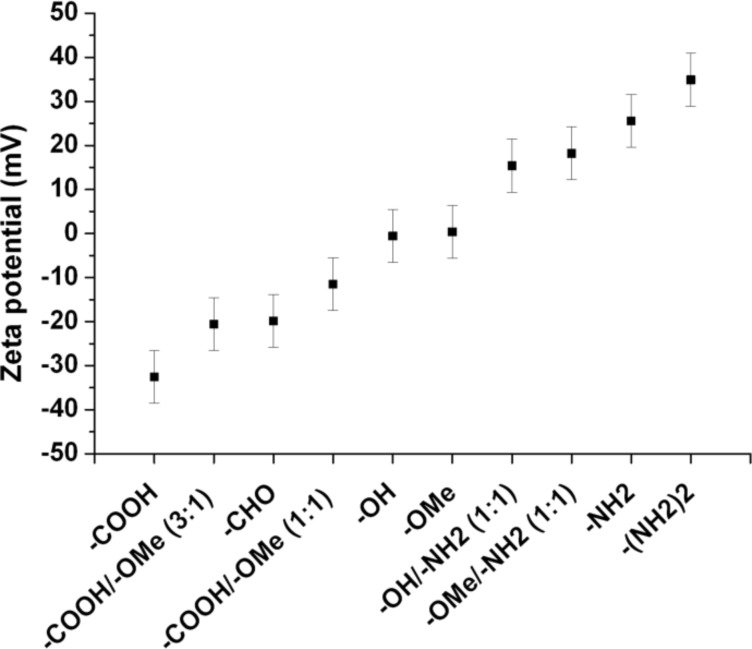
Zeta potential of PI-*b*-PEG encapsulated QDs with different end groups in deionized water. Reproduced with permission from [30]. Copyright 2013 The Royal Society of Chemistry.

The encapsulation of inorganic nanoparticles like iron oxide particles or QDs in the as functionalized PI-*b*-PEGs did not change the properties of the material, as it has already been observed for regular PI-*b*-PEG. This indicates, that the functional groups embedded on the outer part of the micelle do not interact significantly with the surface of the inorganic NP. In contrast, the surface charge was influenced by the end-group of the polymer as it was expected. This could be proven by zeta potential measurements in deionized water, as can be seen in Figure 8. The charge could be adjusted between −35 and +35 mV by properly mixing neutral and amine- or carboxy-functionalized PI-*b*-PEGs [30].

**Figure 9:**
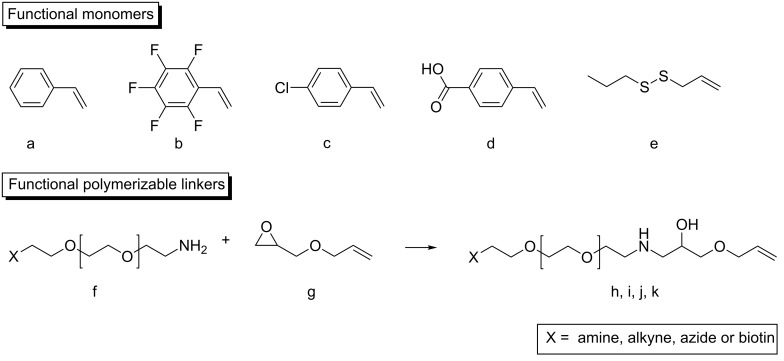
Different applied monomers for the functionalization during the seeded emulsion polymerization (upper part); strategy for the introduction of a PEG linker between the functional group or the functional molecule and a polymerizable alkene function (lower part) [40].

Whereas the functionalization properties of the pure PI-*b*-PEG system are mainly limited to reactions prior to the encapsulation of particles, the seeded emulsion polymerization opens the possibility to functionalize the nanocontainers during the polymerization process. Molecules with an alkene function are suitable to be introduced into the polymeric shell via radical polymerization. Since this reaction takes place in the hydrophobic region of the micelle, small functional monomers will be found there and at the interface between the hydrophobic and hydrophilic part. This can be avoided by using a spacer like PEG between the alkene and the functional molecule (see Figure 9). Combined with the pre-phase transfer functionalization of PI-*b*-PEO a successive emulsion polymerization gives the possibility of adding a variety of functional monomers to the nanoparticle.

### In vitro studies

The influence of differently sized and functionalized nanocontainers on the interaction with cells was tested on human alveolar epithelial cells (A549). First, a general investigation of the toxicity of various nanocontainers (QDs, iron oxide particles, differently sized and functionalized PI-*b*-PEG) was performed, using standard WST8- and LDH-assays. In a region between 0.1–1.0 µmol/L none of the tested constructs showed any toxicity or influence on the viability of the cells [25,30]. The properties of a representative selection concerning the cellular uptake was investigated by incubating A549 cells under biologically relevant conditions for up to 4 h and under more radical conditions in serum free media for 20 h and analyzed via confocal microscopy. The tested samples and the obtained results are summarized in Table 2.

**Table 2:** Tested samples and the observed interaction with A549 cells.

Name	*M*_n_ (PI-*b*-PEG)	Functional group	Regular medium	Medium without serum

Container A-1	4300	Hydroxy	—	Uptake
Container A-2	6600	Hydroxy	—	Uptake
Container A-3	9000	Hydroxy	—	Uptake
Container A-4	10500	Hydroxy	—	Uptake
Container A-5	13400	Hydroxy	—	—
Container B-1	4300	Methoxy	—	Uptake
Container B-2	6600	Methoxy	—	Uptake
Container B-3	9000	Methoxy	—	Uptake
Container B-4	10500	Mehtoxy	—	—
Container B-5	13400	Methoxy	—	Partial uptake
Container C-1	4300	Amino	—	Uptake
Container C-2	6600	Amino	—	Uptake
Container C-3	9000	Amino	—	Uptake
Container C-4	10500	Amino	—	—
Container D-1	4300	Carboxy	—	—
Container D-2	6600	Carboxy	—	—
Container D-3	9000	Carboxy	—	Sticks to the cell membrane
Container D-4	10500	Carboxy	—	Partial uptake
Container D-5	13400	Carboxy	—	Uptake

**Figure 10:**
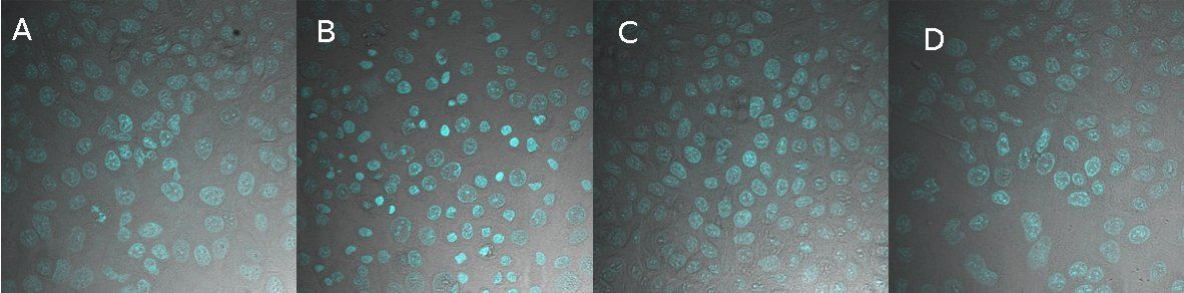
Confocal microscopy images of A549 cells, incubated with different nanocontainers under serum-containing conditions for 4 h. Hydroxy-functionalized PI-*b*-PEG (A); methoxy-functionalized PI-*b*-PEG (B); amine-functionalized PI-*b*-PEG (C); carboxy-functionalized PI-*b*-PEG (D).

None of the samples showed unspecific adhesion or uptake on A549 cells in serum containing medium after 4 h, although the containers range in a suitable size range between 40 and 80 nm for endocytosis [22]. Even the positively charged samples, bearing the amino functions showed no unspecific interaction with the cell membrane, which qualifies the nanocontainers in a first step as versatile tools for specific targeting, since no unspecific background has to be expected. Figure 10 shows exemplarily the confocal microscopy images for the cells incubated with QDs encapsulated in the smallest PI-*b*-PEG with the four different functional groups.

A general trend for the size dependent interaction between non-charged nanocontainers and A549 cells has already been reported [24]. Thereby an upper limit for encapsulated QDs in a PI-*b*-PEG of 13400 g/mol was recognized, since these samples showed no uptake under serum free conditions, while the smaller sized nanocontainers were internalized. The investigation under these conditions was extended to methoxy-, carboxy-, and amine-functionalized particles. Comparable results were obtained for the methoxy functionalized particles. Although the trend was not exactly identical, it clearly can be seen that the uptake for the bigger methoxy nanocontainers does not take place or is less pronounces than for the smaller ones (see Supporting Information File 1, Figure S1). As it was expected, most of the positively charged nanocontainers were taken up easily by the cells (see Supporting Information File 1, Figure S2), except for the biggest sample. This again could be due to a steric hindrance, since the positively charged samples show slightly higher hydrodynamic diameters then their neutral charged counterparts. However, this is in good agreement with literature findings concerning the uptake of positively charged nanoparticles [35,41].

Interestingly, a reverse size-dependent effect for the negatively charged nanocontainers was observed. While the smaller samples showed no interaction with the cells at all, the bigger samples were taken up by the cells (see Supporting Information File 1, Figure S3). However, it has to be kept in mind, that the uptake conditions are extremely harsh.

**Figure 11:**
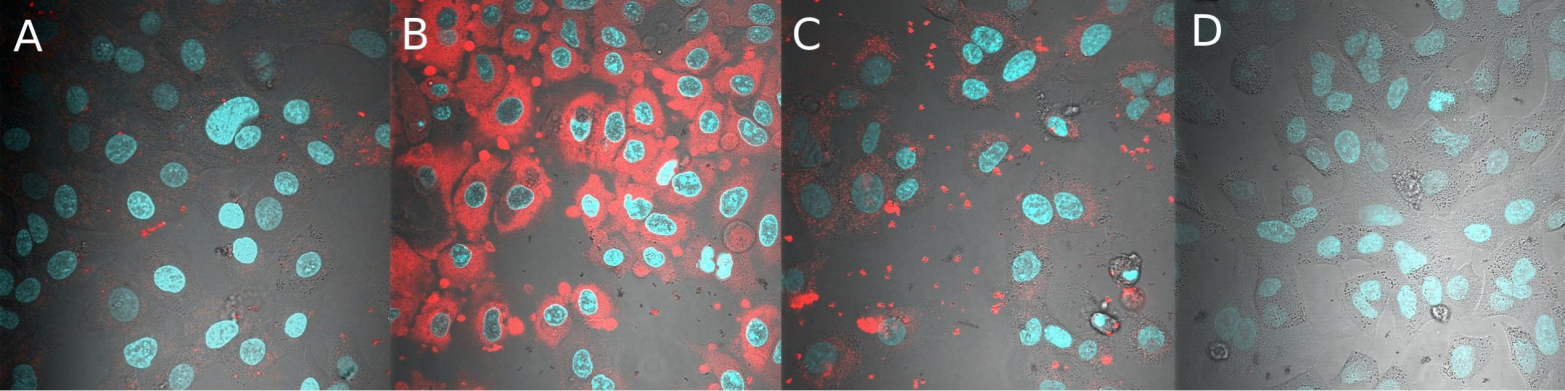
Confocal microscopy images of A549 cells, incubated with different nanocontainers under serum-free conditions for 20 h. Hydroxy-functionalized PI-*b*-PEG (A); methoxy-functionalized PI-*b*-PEG (B); amine-functionalized PI-*b*-PEG (C); carboxy-functionalized PI-*b*-PEG (D).

Figure 11 shows the uptake behavior for the four above shown samples under serum free conditions. While the neutral and positively charged nanocontainers were taken up by the cells, the negatively charged nanocontainers showed no interaction with the cells at all.

From these experiments can be concluded, that the as prepared nanocontainers are suitable for specific targeting attempts, since they on the one hand do not show any unspecific interaction with the cells under default conditions with serum containing medium. On the other hand, the nanocontainers are in a good size range and can in general be internalized by the cells.

First experiments in tumor targeting with antibody coupled QDs and iron oxide particles have already shown this specificity in vivo. While particles coupled with a non-specific antibody or no functionality showed no elongated interaction, the nanocontainers bearing the tumor specific antibody were detected in the tumor tissue even after longer circulation times [25]. These results proof the superior applicability of the PI-*b*-PEG encapsulated nanoparticles in biomedicine.

## Conclusion

This review has shown how the amphiphilic PI-*b*-PEG diblock copolymer can be used as a very versatile and powerful tool for the encapsulation of inorganic nanoparticles for the biomedical use. Especially the easily adjustable properties like size, surface chemistry and the shielding of the nanoparticles within the resulting nanocontainer are of a high importance, since these parameters determine the interaction with biomaterial. Furthermore, it has been demonstrated that this diblock copolymer system fulfills all stated requirements for biomedical applications such as no toxicity and no unspecific interaction in vitro and in vivo. The vast functionalization properties prior to and after the encapsulation of nanoparticles, even including copper catalyzed azide–alkyne cycloaddition make the PI-*b*-PEG nanocontainers a powerful tool for further in vivo experiments in future.

## Supporting Information

File 1 Additional information about the conducted cell culture experiments available.
