# Supplementary material for: Tailoring the ligand shell for the control of cellular uptake and optical properties of nanocrystals
Source: Beilstein J Nanotechnol. 2015 Jan 21;6:232–42. doi: 10.3762/bjnano.6.22 (PMC4311591; doi:10.3762/bjnano.6.22)
Supplement: File 1 — Additional information about the conducted cell culture experiments available. [file Beilstein_J_Nanotechnol-06-232-s001.pdf]

Supporting Information

for

Tailoring the ligand shell for the control of cellular uptake and optical properties of nanocrystals

Johannes Ostermann[1], Christian Schmidtke[1], Christopher Wolter[1], Jan-Philip Merkl[1], Hauke Kloust[1] and Horst Weller*[1,2,3,4]

Address: [1]Institute for Physical Chemistry, University of Hamburg, Grindelallee 117, 20146 Hamburg, Germany, [2]Center for Applied Nanotechnology, Grindelallee 117, 20146 Hamburg, Germany, [3]The Hamburg Centre for Ultrafast Imaging, University of Hamburg, Luruper Chaussee 149, 22761 Hamburg, Germany and [4]Department of Chemistry, Faculty of Science, King Abdulaziz University, Jeddah, Saudi Arabia

Email: Horst Weller - weller@chemie.uni-hamburg.de

* Corresponding author

Additional information about the conducted cell culture experiments available

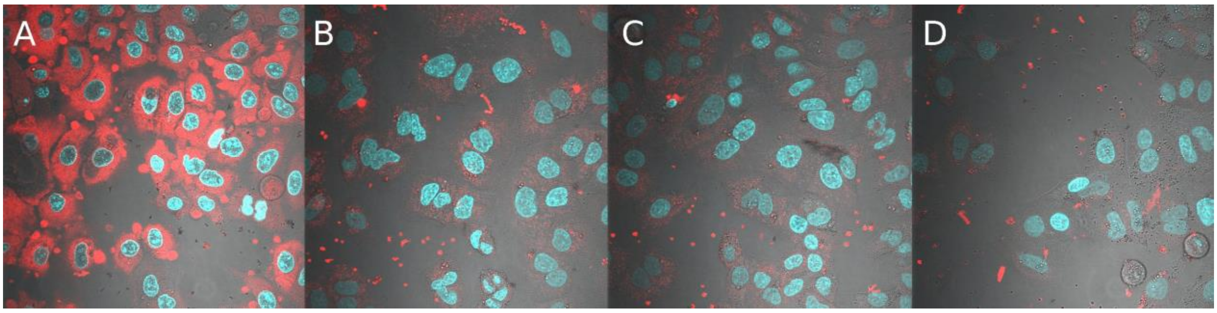

Figure S1: Confocal microscopy images of A549 cells, incubated with methoxy-functionalized nanocontainers under serum-free conditions for 20 h. 4300 g/mol PI-*b*-PEG (A); 6600 g/mol PI-*b*-PEG (B); 9000 g/mol PI-*b*-PEG (C); 13400 g/mol PI-*b*-PEG (D).

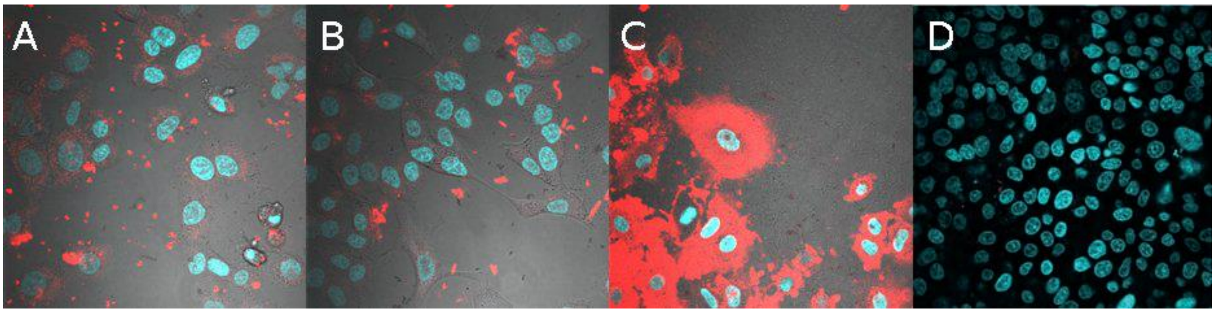

Figure S2: Confocal microscopy images of A549 cells, incubated with amine-functionalized nanocontainers under serum-free conditions for 20 h. 4300 g/mol PI-*b*-PEG (A); 6600 g/mol PI-*b*-PEG (B); 9000 g/mol PI-*b*-PEG (C); 10500 g/mol PI-*b*-PEG (D).

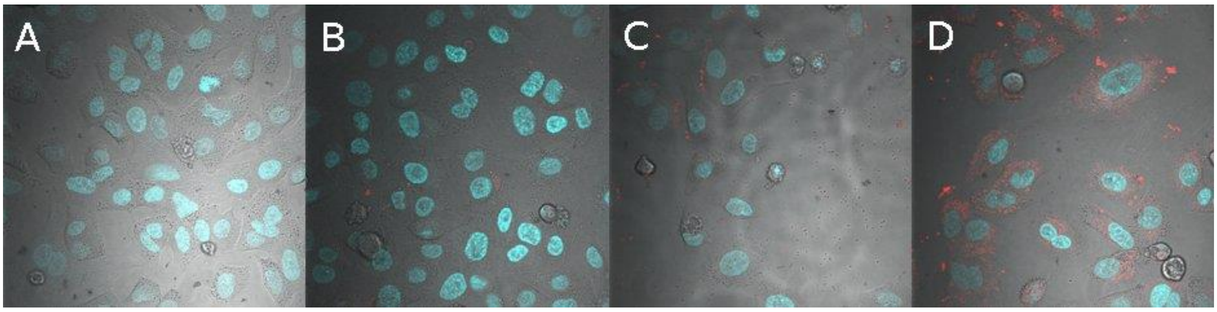

Figure S3: Confocal microscopy images of A549 cells, incubated with carboxy-functionalized nanocontainers under serum-free conditions for 20 h. 4300 g/mol PI-*b*-PEG (A); 6600 g/mol PI-*b*-PEG (B); 9000 g/mol PI-*b*-PEG (C); 13400 g/mol PI-*b*-PEG (D).
